# An Experimental Study on ^131^I-CHIBA-1001: A Radioligand for α7 Nicotinic Acetylcholine Receptors

**DOI:** 10.1371/journal.pone.0070188

**Published:** 2013-07-30

**Authors:** Lei Yin, Qian Zhao, Ling Li, Su Lei Zhang, Xue Qi Chen, Chao Ma, Lei Kang, Meng Liu, Chun Li Zhang, Ping Yan, Rong Fu Wang

**Affiliations:** 1 Department of Nuclear Medicine, Peking University First Hospital, West District, Beijing, China; 2 Department of Nuclear Medicine, General Hospital of Ningxia Medical University, Xingqing District, Yinchuan, Ningxia, China; Northwestern University Feinberg School of Medicine, United States of America

## Abstract

**Objective:**

The α7 nicotinic acetylcholine receptors (nAChRs) play a vital role in the pathophysiology of neuropsychiatric diseases such as Alzheimer’s disease and depression. However, there is currently no suitable positron emission tomography (PET) or Single-Photon Emission Computed Tomography (SPECT) radioligands for imaging α7 nAChRs in brain. Here our aim is to radiosynthesize a novel SPECT radioligand ^131^I-CHIBA-1001 for whole body biodistribution study and in vivo imaging of α7 nAChRs in brain.

**Method:**

^131^I-CHIBA-1001 was radiosynthesized by chloramine-T method. Different conditions of reaction time and temperature were tested to get a better radiolabeling yield. Radiolabeling yield and radiochemical purities of ^131^I-CHIBA-1001 were analyzed by thin layer chromatography (TLC) and high-performance liquid chromatography (HPLC) system. Whole body biodistribution study was performed at different time points post injection of ^131^I-CHIBA-1001 in KM mice. Monkey subject was used for in vivo SPECT imaging in brain.

**Result:**

The radiolabeling yield of ^131^I-CHIBA-1001 reached 96% within 1.5∼2.0 h at 90∼95°C. The radiochemical purity reached more than 99% after HPLC purification. ^131^I-CHIBA-1001 was highly stable in saline and fresh human serum in room temperature and 37°C separately. The biodistribution data of brain at 15, 30, and 60 min were 11.05±1.04%ID/g, 8.8±0.04%ID/g and 6.28±1.13%ID/g, respectively. In experimental SPECT imaging, the distribution of radioactivity in the brain regions was paralleled with the distribution of α7 nAChRs in the monkey brain. Moreover, in the blocking SPECT imaging study, the selective α7 nAChR agonist SSR180711 blocked the radioactive uptake in the brain successfully.

**Conclusion:**

The CHIBA-1001 can be successfully radiolabeled with ^131^I using the chloramine-T method.^ 131^I-CHIBA-1001 can successfully accumulate in the monkey brain and image the α7 acetylcholine receptors. ^131^I-CHIBA-1001 can be a candidate for imagingα7 acetylcholine receptors, which will be of great value for the diagnosis and treatment of mental diseases.

## Introduction

Neuronal nicotinic acetylcholine receptors (nAChRs), which are members of the four transmembrane domain superfamily of neurotransmitter-gated ion channels, are pentameric combinations of α and α/β subunits, with a high degree of complexity conferred by 12 different α (α2–α10) and β (β2–β4) subunits [1]. Although neuronal nAChRs play an array of critical roles in the central nervous system (CNS), only in the last two decades a rapid growing understanding of subtype localization has been associated with potential therapeutic applications. Indeed, the most abundant nAChR subtypes in the CNS are α4β2 heteromers and α7 homomers. The α4β2 subtypes are expressed predominantly in the cortex, hippocampus, visual cortex, striatum and substantia nigra, mesocorticolimbic pathway, and nucleus raphe magnus, whereas the α7 subtypes are mainly localized in the cortex, hippocampus, and auditory cortex [2]. Functionally, α7 channels are easily distinguished from α4β2-containing receptors due to their lower affinity for acetylcholine, a high affinity toward a-bungarotoxin, a rapid desensitization, and a relatively high permeability to calcium [2], [3]. And α7 nicotinic acetylcholine receptors (α7 nAChRs) are currently being investigated as a potential therapeutic target for cognitive disturbances in schizophrenia and Alzheimer disease based on data from small open clinical studies [4]. Over the last decade, further evidence of such effects has been generated in animal models showing that systemic administration of small-molecule α7 nAChR partial agonists produce effects on several domains of cognition [5]. Recently, animal studies using α7 nAChR knockout mice have demonstrated that α7nAChRs might be involved in mediating the attentional effects of nicotine [6], [7]. Hence, access to a suitable radiolabeled α7 nAChR tracer is very important in research on CNS. Several α7 nAChR-selective radioligands such as PNU- 282987 [8], [9],PHA-543613 [10],AR-R17779 [11],SSR180711[12]–[15] and A-582941 [16] have already been tested in animal and human studies. It is therefore likely that α7 nAChR agonists will be useful as therapeutic drugs for cognitive deficits in several neuropsychiatric diseases [17].

However, there have been few reports on radioiodine-labeled radioligands based on α7 nAChR. The use of the above-described radiolabeled compounds for in vivo studies is limited at the present time because of their high nonspecific binding affinity and poor blood-brain barrier (BBB) permeability [18].

Here, we developed a novel iodine-131 labeled radioligand, ^131^I-CHIBA-1001, a radioiodine-derivative of SSR180711 for the first time, and characterized its binding affinity to α7nAChRs in the rat brain. In addition, monkey studies were used to evaluate the ligand for in vivo SPECT imaging of α7 nAChRs.

## Materials and Methods

### Ethics Statement

This study was carried out in strict accordance with the recommendations. All animal experiments were approved by Peking University Animal Studies Committee, according to the Guidelines for the Care and Use of Research Animals (Peking University, China) (Approval ID: J201133, J201134). All animals, housed and handled in strict accordance with good animal practice under supervision of veterinarians, received environmental enrichment and were monitored for evidence of disease and changes in attitude, appetite, or behavior suggestive of illness. In accordance with the recommendations of Weatherall report, “The use of non-human primates in research,” every effort was made to alleviate animal discomfort and pain by appropriate and routine use of anesthetic and/or analgesic agents. The rhesus subjects scanned in this study were pair-housed to promote normal interactive behavior. Food and water was readily available for the monkeys. The brain images used in this study were acquired non-invasively using SPECT technology. The only invasive procedure performed was the establishment of a venous catheter for the administration of anesthetics during image acquisition. Catheter placement was performed under aseptic conditions, and animals were monitored continuously during anesthesia to assure maintenance of normal physiological parameters. Animals were fasted starting the night prior to the procedure to reduce risk of aspiration. After initial intramuscular administration of 15 mg/kg ketamine, an intravenous line was established and used to initiate anesthesia using midazolam (0.25 mg/kg) and ketamine (5 mg/kg). Each animal was also given 0.027 mg/kg atropine and intubated to protect the airway and reduce risk of aspiration. After intubation, respiratory rate was monitored frequently and used to maintain adequate anesthesia using midazolam and ketamine. After the SPECT procedure, anesthesia was discontinued and all animals were directly observed until they recovered completely from the anesthesia and returned to normal functioning. All animals tolerated the procedure well and there were no adverse events associated with any of the experiments.

### General

**Figure 1:**
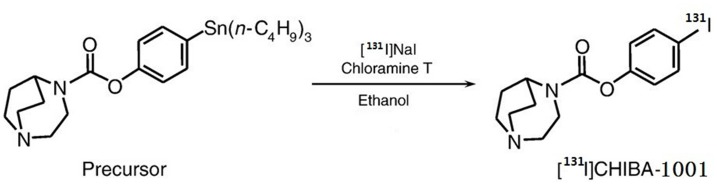
Synthesis of ^131^I-CHIBA-1001.

CHIBA-1001, its precursor, 4-(tributylstannyl) phenyl 2,5-diazabicyclo [3.2.2] nonane-2-carboxylate (Fig. 1) and SSR180711 were kindly provided by Professor Kenji Hashimoto (Chiba University Center for Forensic Mental Health), and the synthesis methods were described in their paper [19]. The solution of acetic acid ethanol was freshly prepared just before use.

### Radiosynthesis of ^131^I-CHIBA-1001

^131^I-CHIBA-1001 was radiosynthesized by the chloramine-T method with the precursor and Na^131^I. The precursor (50 µg) was dissolved in 250 µL of 1% acetic acid ethanol solution. A precursor ethanol solution (250 µL) and 200 mM chloramine-T ethanol solution (7 µL) were added to 50 µL of Na^131^I. The reaction temperature and duration of reaction mixture were set as 70, 80, 90 and 100°C for 30, 60, 90 and 120 min, respectively.

### Thin Layer Chromatography Analysis

Radiolabeling yields of ^131^I-CHIBA-1001 were determined using thin layer chromatography (TLC) analysis. TLC was performed using silica gel GF-254 coated on glass plates (Analtech Inc., Newark, DE). For radiochemical analysis, the plates were marked into 10 equal sections. After development and drying, the plates were cut into sections, and the radioactivity of each section was counted in a gamma well counter.

### High Performance Liquid Chromatography Analysis

Radiochemical purities of ^131^I-CHIBA-1001 were determined by HPLC analysis. HPLC analysis was performed with a Venusil MP C-18 column (10 mm×250 mm, 5 µm) by using an Shimadzu System with SCL-10AVP HPLC pump. The radioactive peak fraction eluted by CH_3_CN/50 mM CH_3_COONH_4_/50 mM CH_3_COOH = 250/375/375 at a flow rate of 3 ml/min was collected into an evaporation flask and evaporated to dryness, and the residue was re-dissolved with 2 ml of ethanol, the solution was passed through a 0.22 µm pore size filter before intravenous administration to the monkey. Chemical and radiochemical purities of ^131^I-CHIBA-1001 were analyzed by HPLC system consisting of a Alltima C-18 column (250 mm×4.6 mm,5 µm) with CH_3_CN/50 mM CH_3_COONH_4_/50 mM CH_3_COOH = 250/375/375 as a mobile phase at a flow rate of 1 ml/min.

### In Vitro Stability Analysis

To evaluate the in vitro labeling stability, the radiochemical purity was determined by incubating ^131^I-CHIBA-1001 with normal saline at 4°C and freshly collected serum at 37°C, respectively. The aliquots were then analyzed at 1 h, 3 h, 6 h, 24 h and 48 h by HPLC.

### In Vitro Binding Assays

Male SD (Sprague Dawley) rats (8–10 week olds, 180–200 g) were used for the experiments. After sacrificing the rats by decapitation, the brains were rapidly removed from the skulls.

The tissues of each brain region were homogenized in 15 volumes of 50 mM Tris–HCl buffer (120 mM NaCl, 2 mM KCl, 1 mM CaCl_2_, 1 mM MgCl_ 2_, pH 7.4 at 4°C) using a Polytron homogenizer at setting No. 5 for 30s on ice. The membranes were centrifuged in 50 ml polypropylene tube at 48,000 g for 20 min at 4°C. The supernatant was discarded and the pellet re-suspended, homogenized and centrifuged as above. The membrane pellet was washed and re-suspended in ice-cold buffer and was then centrifuged two more times. The final pellet was re-suspended in 10 volumes of the same buffer. The protein concentrations were measured according to the method of Lowry [20].

Assays of the binding of ^125^I-CHIBA-1001 to α7 nAChRs in the rat brain were performed according to the method published previously [21], [22]. The reaction mixtures were consisted of aliquots of a membrane suspension (200 µl), ^125^I-CHIBA-1001 and the indicated concentrations of test drug in a final volume of 0.5 ml in duplicate. Non-specific binding was estimated in the presence of 30 µM SSR180711. Binding reaction was conducted for 150 min at 4°C for the equilibrium saturation and inhibition studies.

### Biodistribution Study

40 male KM mice (8–10 week olds, 15–30 g) were randomly divided into 8 groups of 5 mice each. Each mouse was injected with 1 µg (1,850 kBq) of ^131^I-CHIBA-1001 in 250 µl of normal saline via the lateral tail vein. At 15 min, 30 min, 1, 2, 4, 6, 8 and 24 h, 5 mice of each group were sacrificed by cervical dislocation after 100 µl of blood samples were collected. Tissues of interest (heart, liver, spleen, lung, kidney, stomach, small intestine, bladder, skeletal muscle, bone marrow, and brain) were removed and weighed. Radioactivity of all tissues was measured with a NaI (Tl) well counter. Biodistribution results were recorded as percentage of injected dose per gram (%ID/g),calculated by tissue mass and radioactivity.

### γ-Camera Imaging

Four monkeys (male, 7 year olds, 8 kg) were selected for SPECT imaging. Immediately after HPLC column purification, 50 µg (74 MBq) of the ^131^I-CHIBA-1001 in 2 mL of normal saline were injected into the monkeys via intravenous administration after administration of saline (control) or SSR180711 (5.0 mg/kg, i.v.). All injections were tolerated well. At 0.5, 1.5, and 2 h after injection, imaging was performed in the Department of Nuclear Medicine, Peking University First Hospital, using SPECT (SPR SPECT; GE Healthcare, Inc.) equipped with a high-energy general purpose (HEGP) collimator. Static images (200,000 counts), obtained with a zoom factor of 2.0, were digitally stored in a 64 × 64 matrix.

### Statistical Analysis

The software SPSS 17.0 was used. Variables are expressed as average ± SD. Statistical comparisons of variables were performed by ONE-WAY ANOVA analysis. P values of less than 0.05 were considered statistically significant.

## Results

### Radiosynthesis of ^131^I-CHIBA-1001

**Figure 2:**
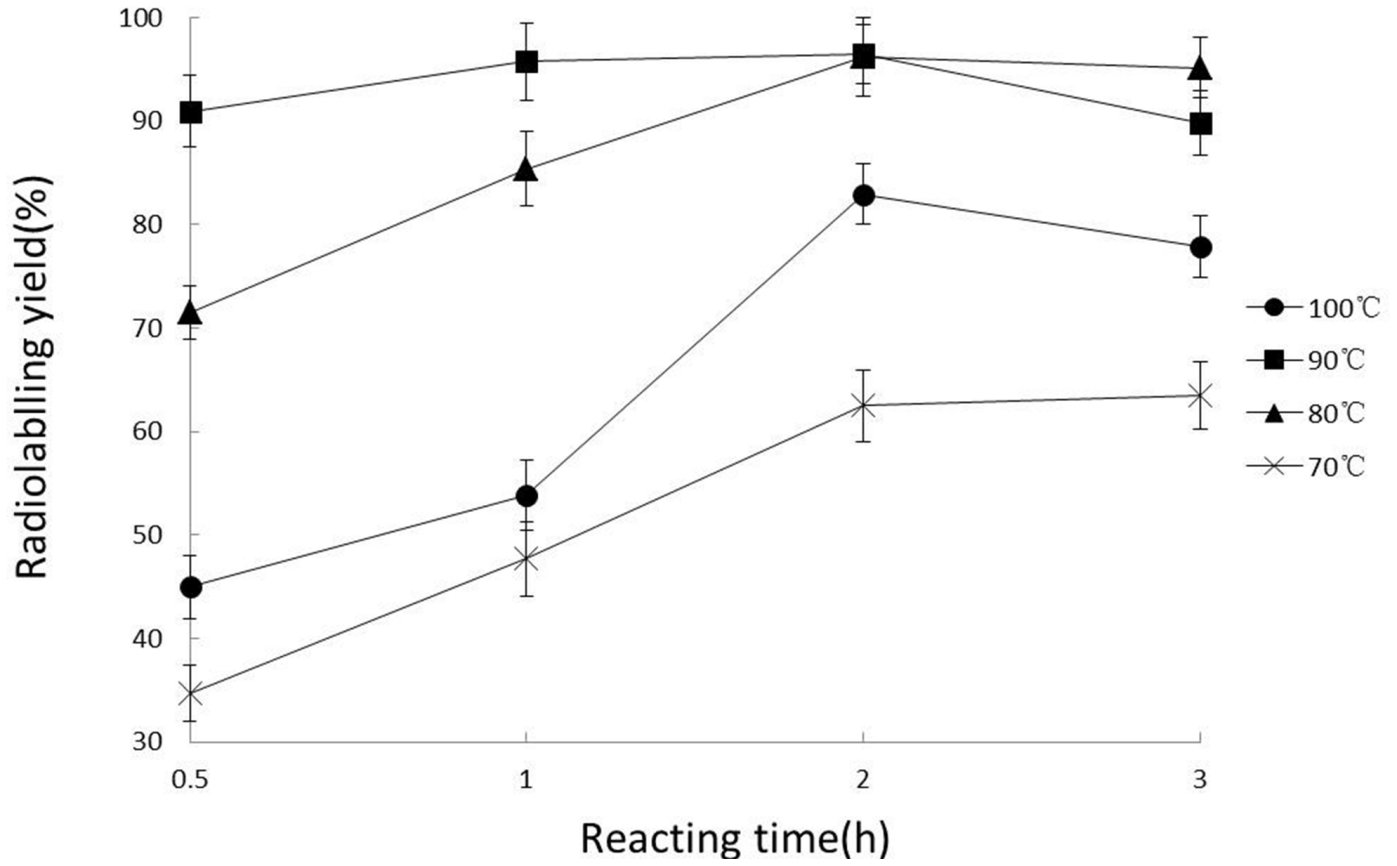
Radiolabeling yields of ^131^I-CHIBA-1001 with different conditions.

TLC was used to monitor the radiolabeling yields of ^131^I-CHIBA-1001. It was found that the reaction time was very important to the success of the radiosynthesis. Radiolabelling yield increased with the prolonged reaction time. However, when the reaction time was more than 2 h, the yield dropped. Moreover, the optimized reaction temperature was between 90 and 95°C.Less than 90°C or more than 95°C of the reaction temperature caused a lower radiolabeling yield (Fig. 2). Therefore, reaction temperature of 90∼95°C and reaction duration of 90∼120 min were selected as the best radiolabeling conditions. The radiolabeling yield was high as 96.5% ±2.8% under the conditions.

**Figure 3:**
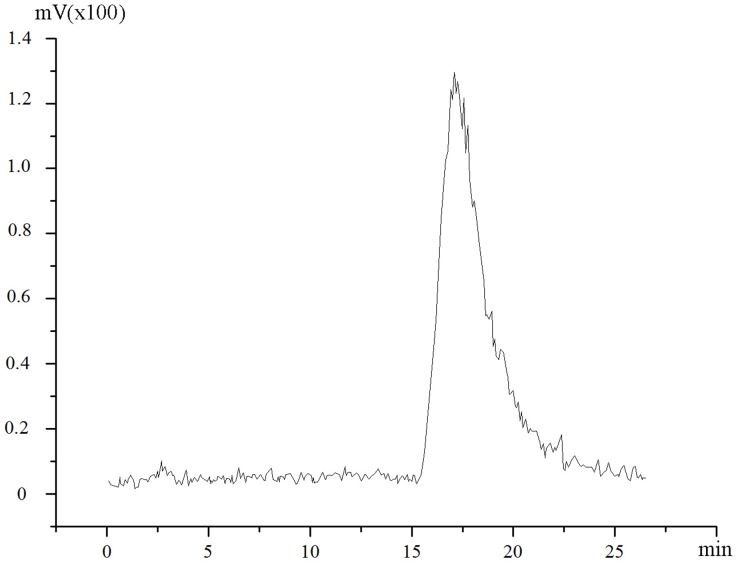
HPLC chromatograms of ^131^I-CHIBA-1001 performed with a Alltima C-18 column (250 mm×4.6 mm, 5 µm). HPLC = high-performance liquid chromatography.

HPLC was employed to calculate the purity of the radiolabeled compounds. The reaction mixture was diluted with the HPLC mobile phase and then purified by HPLC. HPLC analysis revealed that the yield of ^131^I-CHIBA-1001 was 96.5% ±3.2%. Results of HPLC analysis showed that only one single peak was observed (Fig. 3). This clearly showed that the complexes were pure with no residual Na^131^I or other impurities. Results of both TLC and HPLC showed that radiolabeling yields of ^131^I-CHIBA-1001 was more than 96%. Hence, these radiolabeled compounds could be used immediately without further purification for both in vitro and in vivo studies.

**Figure 4:**
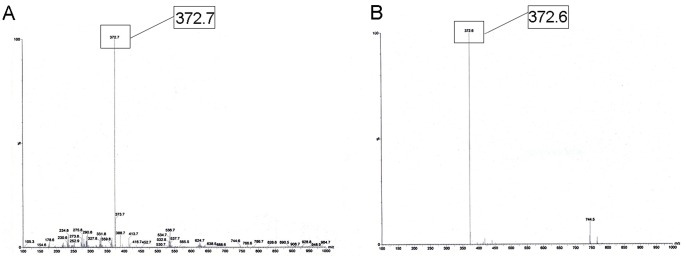
Mass spectroscopy analysis of ^127^I-CHIBA-1001(A) and ^127^I-CHIBA-1001 standard substance (B).

Mass spectroscopy (MS) analysis showed that the molecule weight (MW) of ^131^I-CHIBA-1001 was 372.7, which was correlated with the MW of ^127^I-CHIBA-1001 standard substance offered by Chiba University Center for Forensic Mental Health. (Fig. 4).

### In Vitro Stability Analysis

After CHIBA-1001 was radiolabeled with ^131^I under optimal conditions, HPLC was used in stability analysis. The radiochemical purity of ^131^I-CHIBA-1001 was more than 99% and highly stable within 48 h. Radiolabeled CHIBA-1001 exhibited no significant degradation and off-labeling. No significant trend for the radiochemical purity to be lower in fresh human serum than in normal saline was observed during the incubation. It was shown that radiolabeled CHIBA-1001 had a good tolerance in serum at 37°C, which was similar to in vivo the condition.

### Biodistribution Study

**Figure 5:**
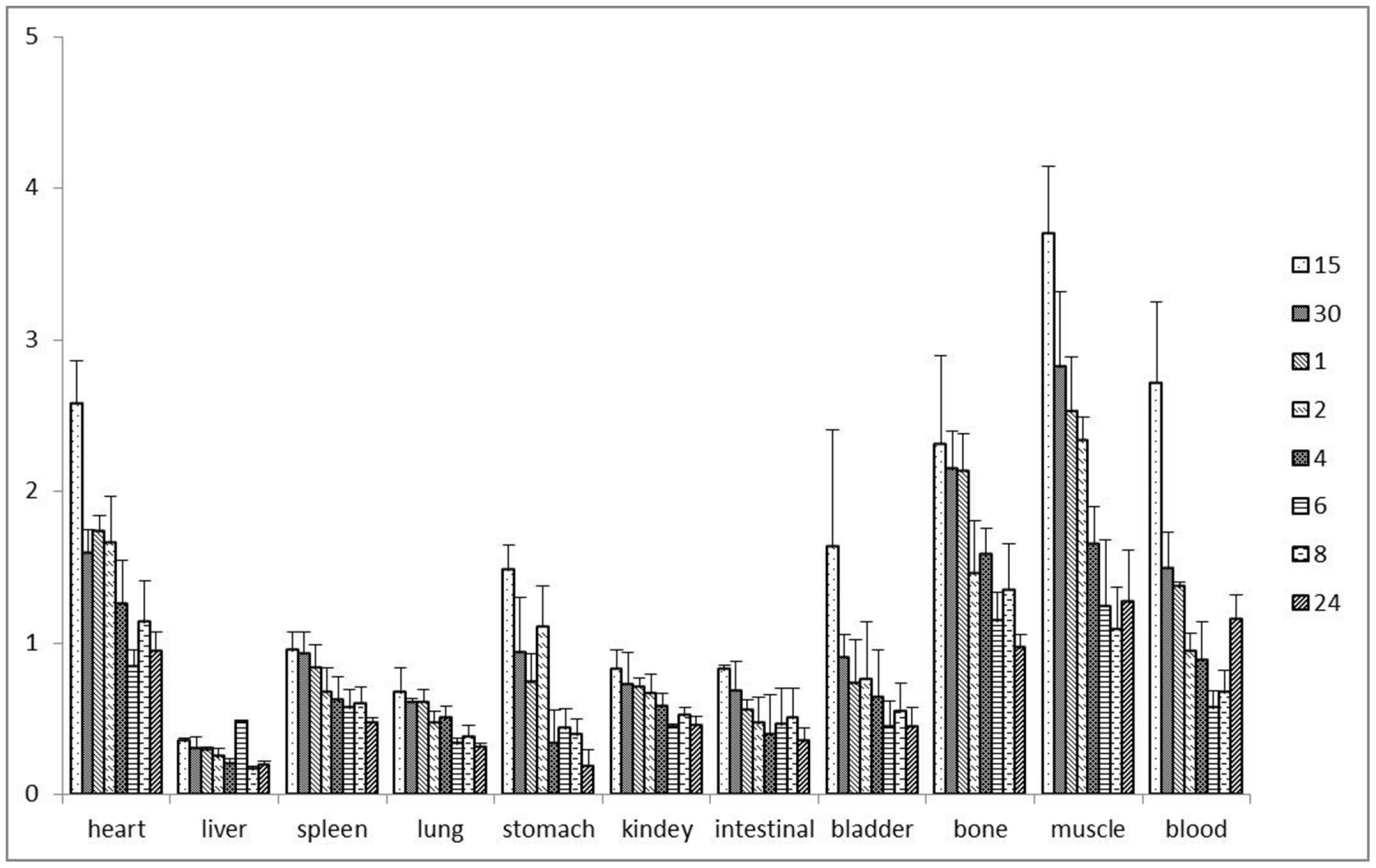
Brain-to-nonbrain ratios of different tissues in normal mice 15 m, 30 m, 1, 2, 4, 6, 8 and 24 h after injection of ^131^I-CHIBA-1001. Brain-to-nonbrain ratios were calculated from data in Table 1.

Biodistribution results of ^131^I-CHIBA-1001 were shown in Table 1. At different time points after injection of ^131^I-CHIBA-1001, the liver showed the highest initial uptake followed by the lung, kidneys, small intestines, spleen, brain, and bladder. The liver and kidney uptake reached 31.69±3.64%ID/g and 13.6±2.04%ID/g, respectively. Furthermore, the biodistribution data of brain at 15, 30, and 60 min were 11.05±1.04%ID/g, 8.8±0.04%ID/g and 6.28±1.13%ID/g, respectively. At 60 min the retention of radiolabeled ligand in the brain was up to 50% of the 15 min uptake. Hence, the ratio of brain-to-blood and brain-to-muscle uptake after injection of ^131^I-CHIBA-1001 were significantly higher (P<0.05), especially at 15 min.The ratio of brain-to-blood and brain-to-muscle at 15 min reached 2.7 and 3.7, respectively. (Fig. 5).

**Table 1:** Biodistribution (%ID/g) of ^131^I-CHIBA-1001 in mice.

Tissue	15 min	30 min	1 h	2 h	4 h	6 h	8 h	24 h
heart	4.34±0.73	4.32±0.95	3.63±0.7	2.68±0.85	2.25±0.77	1.39±0.42	0.86±0.33	0.64±0.12
liver	31.69±3.64	24.99±3.83	21.56±2.76	16.75±1.88	13.43±1.95	6.07±3.4	5.93±2.51	3.79±1.8
spleen	11.85±2.03	8.23±2.98	6.56±0.6	5.81±1.81	4.64±1.68	2.13±0.82	1.71±1	1.55±0.71
lung	16.54±2.66	12.13±3	10.5±2.48	7.71±1.05	5.5±1.61	3.49±0.77	2.54±0.9	2.33±0.86
stomach	7.5±0.83	8.44±1.79	7.36±1.22	5.28±0.51	4.61±0.17	2.35±0.41	1.69±0.15	3.63±2.06
kindey	13.6±2.04	10.53±2.12	8.83±1.05	5.43±1.34	4.64±0.78	2.62±0.59	1.49±0.18	1.55±0.49
intestinal	13.46±0.98	11.09±2.85	11.3±1.95	9.65±1.58	9.7±1.55	2.48±0.68	2.01±0.82	2.07±0.69
bladder	5.31±2.14	8.33±2.02	7.82±2.37	6.74±2.61	4.74±1.64	3.02±1.82	1.97±0.96	1.7±0.72
bone	5.11±1.68	3.46±0.93	2.92±0.27	2.46±0.42	1.7±0.34	1.03±0.31	0.73±0.3	0.76±0.36
muscle	2.99±0.26	2.31±0.46	2.58±0.88	1.98±0.26	1.62±0.23	1±0.29	0.92±0.38	0.43±0.13
brain	11.05±1.04	8.8±0.04	6.28±1.13	3.72±0.31	2.71±0.72	1.16±0.31	0.74±0.03	0.72±0.3
blood	4.26±1.18	5.19±0.64	4.59±0.84	4.6±0.98	3.4±0.72	2.06±0.49	1.45±0.52	1.81±0.43

Each value represents average of 5 mice ± SD and is expressed as %ID radioactivity per gram organ or tissue.

%ID/g = injection dose/g organ or tissue (%).

### In Vitro Binding Assays

In saturation binding isotherms, nonlinear regression analysis of specific binding revealed an apparent Kd of 81.87±7.95 nM (95% confidence interval: 73.62 to 89.49 nM) and a B max of 63.97±17.45 fmol/mg protein (95% confidence interval: 46.57 to 81.47 fmol/mg protein) (n = 5, mean ± S.E.M.) at 4°C.

### γ-Camera Imaging

**Figure 6:**
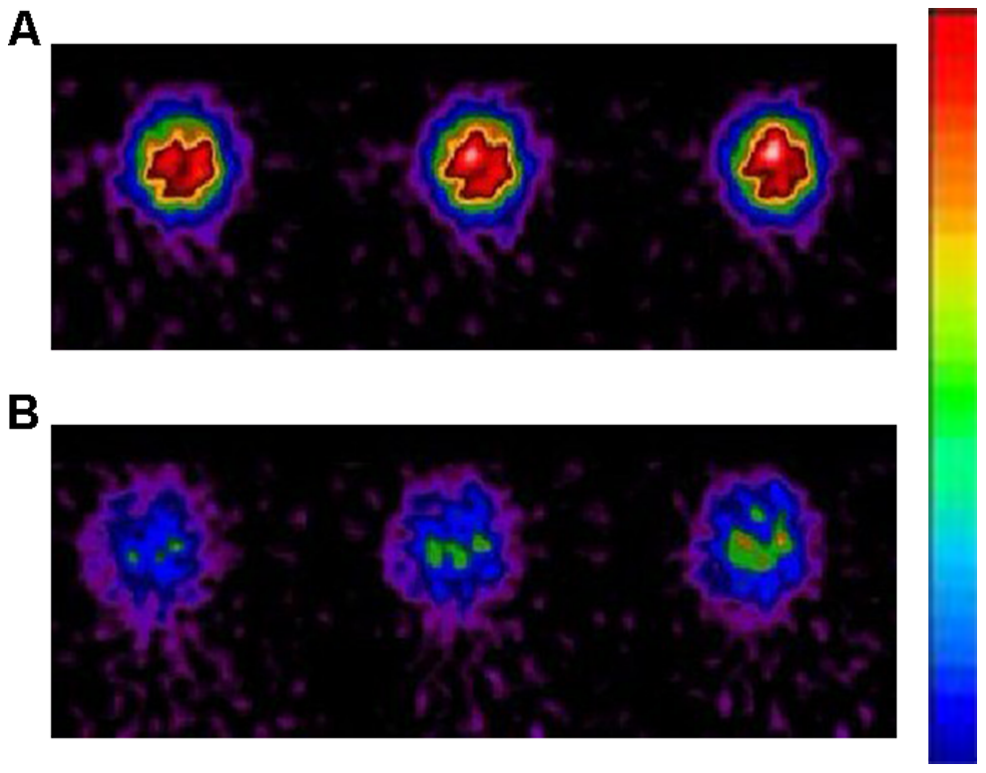
Representative SPECT images in the brains of monkey after intravenous administration of ^131^I-CHIBA-1001. A: Control monkey (saline pre-treated). B: Pretreatment with SSR180711 (30 min before).SPECT = Single-Photon Emission Computed Tomography.

SPECT imaging showed rapid brain penetration and accumulation of ^131^I-CHIBA-1001. The peak time of radioactivity in the brain (occipital cortex, temporal cortex, frontal cortex, thalamus, and cerebellum) was about 30 min after administration of the radioligand. The distribution of radioactivity in the occipital cortex, temporal cortex, frontal cortex and thalamus 30–60 min after administration of the radioligand was higher than that in the cerebellum, consistent with the distribution of α7 nAChRs in the monkey brain. Uptake of radioactivity in the brain after intravenous administration of ^131^I-CHIBA-1001 was decreased by pretreatment with SSR180711 (5.0 mg/kg). (Fig. 6) The blockade in the cerebellum was minimal since the known density of a7-nAChRs is low in this region [23].When the specific binding was estimated by using the radioactivity concentration in the blocked cerebellum as a measure of nonspecific binding, the decrease in the frontal cortex was 38.5±2.1% and the decrease in the thalamus was 35.7±2.8%.

## Discussion

In previous study, Kenji Hashimoto has developed a PET ligand, ^11^C-CHIBA-1001, and observed good imaging effects in the non-human primate brain PET imaging [19].In this study, we firstly report the radiosynthesis, biodistribution, binding experiments and in vivo SPECT imaging of ^131^I-CHIBA-1001. We expected that CHIBA-1001 radiolabeled with I-131 can be used as a SPECT agent.

The characteristics of radiolabeled agents are the key points of preclinical and clinical applications. Better radiolabelling yield, radiochemical purity and specific activity are always the pursuit of the researcher in the field of nuclear medicine. In this study we evaluated the radiolabeling yield under different conditions of reaction time and temperature. Fortunately, the optimal result of radiolabelling yield and the radiochemical purity was 96.5% and 99%, which was much better than previous report [24]. The higher radiolabelling yield provides the advantage for clinical application because of no need for purification.

[^125^I]α-bungar-otoxin [25], [26] and [^125^I]methyllycaconitine ([^125^I]MLA) [27] are well known for researchers who pursued the development of radioligands for α7 nAChRs. Both radioligands have been widely used for studying α7 nAChRs in vitro due to their binding α7 nAChRs irreversibly and selectively in the brain [28], [29]. In our binding studies, the Kd values for ^125^I-CHIBA-1001 binding showed slightly lower overall affinity in rat brain homogenates compared to ^125^I-α-bungarotoxin [30] and^125^I-MLA [27] (Kd = 70 nM vs. 1.5 and 1.8 nM, respectively), however, the Bmax values were in general agreement with the corresponding values determined by both of the above ligands (Bmax = 73 fmol/mg protein vs. 63 and 68 fmol/mg protein, respectively). These results suggest that ^125^I-CHIBA-1001 binding might be similar to ^125^I-α-bungarotoxin binding or ^125^I-MLA binding to α7 nAChRs in the brain of rats.

Expression of α7 nAChRs by epithelial and endothelial cells in the lung has been observed, including primary cultures of human bronchial cultured epithelial cells that have been tested to express α7 mRNA and α-bungarotoxin binding that is indicative of receptor expression [31].High expression of α7 nAChRs by liver macrophages cells has also been proved [32]. Interestingly, the biodistribution of ^131^I-CHIBA-1001 in mice showed that the radiolabeled probes preferentially accumulated in the liver and lung, indicating there were high expression of α7 nAChRs on liver and lung. A number of evidences indicated that cells other than neurons throughout the body expressed nicotinic receptor subtypes, including lymphocytes, macrophages, dendritic cells, adipocytes, keratinocytes, endothelial cells, and epithelial cells of the intestine and lung [33]. In addition, acetylcholine can interact with alpha7 nicotinic acetylcholine receptors expressed by macrophages and other cytokine-producing cells in organs, including the lungs, spleen, liver, kidneys, and gastrointestinal tract [34]. Therefore, this is possible a key reason that there were highly uptake in spleen, stomach, intestines and kidneys. Moreover, the high accumulation in kidneys in this study also indicated that the tracer was cleared probably through the urinary system, whose molecular weight is below the threshold that can be filtered by the glomerular membrane (<60 kDa). High uptake in above organs was a general problem for radiometal-labeled oligonucleotides. This was also an apparent obstacle for imaging α7 nAChRs in these organs. However, it wasn’t a problem for SPECT of α7 nAChRs expression in the brain in our study, where brain was anatomically well separated from the kidneys and liver. The clearance of the radioactivity was slow from the blood, which would favorably influence uptake by brain. The peak time of uptake in brain was about 15 min after administration of the radioligand, with 11.05±1.04%ID/g remaining, which is consistent with the SPECT imaging of ^131^I-CHIBA-1001 in the monkey brain. Furthermore, the uptake in brain decreased rapidly, with 0.74±0.03%ID/g remaining 8 h after injection, which suggested the safety of ^131^I-CHIBA-1001 in brain. The value of %ID/g of all of the organs was significant difference between groups (P<0.05), except for liver. The ratio of brain-to-blood was significant difference (F = 5.35, P<0.05) between groups, especially the highest ratio value appeared at 15 min reached 2.7. The highest ratio of brain-to-skeletal muscle reached 3.7, appeared at 15 min. The high brain/non-target tissue ratios indicated ^131^I-CHIBA-1001 has potential to be a candidate for imaging mental diseases in vivo noninvasively.

Monkey brain is closer to human brain, however, there were few reports on PET or SPECT imaging in the monkey brain based on α7 nAChR. In this study, we first used radioiodine-labeled radioligands in the monkey for SPECT imaging. The results of imaging of α7 nAChRs expression in the monkey brain were consistent with the results of biodistribution data of mice. The peak time of radioactivity in the monkey brain was similar to the peak time of uptake in the mice brain. In addition, blocking imaging studies with excessive the selective α7 nAChR agonist SSR180711 demonstrated specific affinity of the radioligands with α7 nAChR. It is obviously that the SSR180711 blocked the radioactive uptake in the brain successfully. However, an effective half life of the tracer is needed for further study.

Taken together, we have demonstrated that ^131^I-CHIBA-1001 was a novel SPECT ligand for in vivo imaging of α7 nAChRs in the brain. First, the radiolabeling via isotopic exchange did yield a high specific activity of the tracer. The radiolabeling yield of ^131^I labeled CHIBA-1001 was about 96%, and the radiochemical purity is >99%, which indicated that it can successfully detect α7 nAChRs in the brain as a molecular probe. Second, the biodistribution study shows that ^131^I-CHIBA-1001 accumulated in the brain to a level of approximately 11.05%ID/g–a higher level than blood and muscle (The ratio of brain-to-blood and brain-to-muscle at 15 min were 2.7 and 3.7, respectively). This suggested that ^131^I-CHIBA-1001 was suitable for SPECT imaging. Third, an in vivo SPECT study using monkey demonstrated a high accumulation in the brain after intravenous administration of ^131^I-CHIBA-1001. Moreover, the uptake of radioactivity in the monkey brain regions was blocked by pretreatment with the selective a7 nAChR agonist SSR180711.The regional distribution of radioactivity in the monkey brain after intravenous administration of ^131^I-CHIBA-1001 was consistent with the distribution of α7 nAChRs in the monkey brain as reported [35]–[37].

### Conclusion

In summary, ^131^I-CHIBA-1001 can be successfully synthesized using the chloramine-T method with high purity (99%). Biodistribution of ^131^I-CHIBA-1001 showed a quick blood clearance and excretion by the kidneys and bladder. Furthermore, ^131^I-CHIBA-1001 can successfully accumulate in the brain. Hence, the ^131^I labeled CHIBA-1001 would be a useful SPECT molecular probe for the in vivo imaging of α7nAChRs in the human brain.
